# A Rare Case of Fabry’s Disease-Induced Cardiomyopathy: A Case Report and Review of the Literature

**DOI:** 10.7759/cureus.67612

**Published:** 2024-08-23

**Authors:** Ahmed Hussein, Aya Salih, Yousif Mohamed, Emma Thames, L. Maximilian Buja

**Affiliations:** 1 Pathology and Laboratory Medicine, University of Texas at Houston, Houston, USA; 2 Internal Medicine, University of Khartoum, Khartoum, SDN; 3 Radiology, University of Khartoum, Khartoum, SDN; 4 Pathology and Laboratory Medicine, The University of Texas Health Science Center, Houston, USA

**Keywords:** myeloid and curvilinear bodies, biopsy, electron microscope, cardiomyopathy, fabry's disease

## Abstract

Fabry’s disease, also known as Anderson-Fabry Disease (AFD), is caused by mutations in the α galactosidase A (α GalA) gene found on the X chromosome. This condition results in an accumulation of sphingolipids, including globotriaosylceramide (Gb3), in cells throughout the body. The main effects of Fabry disease typically involve heart, kidney, and nervous system complications. A common cardiac dysfunction is left ventricular hypertrophy. In this case study, we share findings about cardiomyopathy resulting from Fabry disease to explain how this condition impacts the heart and the importance of a biopsy in making a diagnosis. A 57-year-old woman with end-stage renal disease likely attributed to hypertension was evaluated for a kidney transplant. An echocardiogram revealed severe ventricular hypertrophy. The clinical team ordered blood levels of alpha-galactosidase, globotriaosylceramide, and globotriaosylsphingosine enzymes, which demonstrated significant deficiency. Consequently, a genetic test along with an endomyocardial biopsy (EMB) was ordered. Under microscopy using hematoxylin and eosin stain (H/E) and periodic acid Schiff stain (PAS), myocyte vacuolization was observed, which remained unchanged when diastase was added. Electron microscopy revealed inclusion bodies described as myeloid and curvilinear bodies within cells, interstitial cells, and cardiomyocytes. Diagnosing Fabry disease can be challenging, as it may be confused with other medical conditions. Our case study showed how EMB played a role in diagnosing the disease and guiding proper treatment.

## Introduction

Fabry’s disease is caused by the deficiency of alpha-galactosidase, an enzyme, leading to the buildup of glycosphingolipids in parts of the body. While this disorder mainly affects males, females can also show symptoms, although they are typically slower to progress and less severe [1,2]. The heart is commonly affected by Fabry disease, causing complications like left ventricular hypertrophy (LVH), right ventricular hypertrophy, conduction disorders, valve malfunctions, and irregular heart rhythms [1]. Cardiac complications are currently one of the main causes of death in individuals with Fabry disease. It’s crucial to distinguish Fabry's disease from conditions that cause LVH, such as hypertrophic cardiomyopathy. This differentiation is important because enzyme replacement therapy (ERT) may help with conditions associated with Fabry’s disease [1-4]. Testing alpha-galactosidase activity levels and examining abnormalities are vital for diagnosing Fabry's disease. It's essential to consider this condition as a diagnosis when patients, especially men, present with unexplained LVH. The buildup of glycosphingolipids in Fabry disease starts before birth. Progresses over time in tissues and organs, including the heart. Heart involvement in Fabry disease often shows up as a type of hypertrophy that is nonobstructive and concentric, which can be a reference point for other conditions with heart muscle thickening [4]. It’s really important to distinguish Fabry's disease from other causes of ventricular hypertrophy because diagnosing it early and starting ERT could potentially help improve or lessen the impact on the heart. Treating Fabry cardiomyopathy usually involves using medications and devices to manage heart-related symptoms. Using ERT like agalsidase alpha or agalsidase beta has been proven to lower the buildup of substances in body tissues, including the heart, which might help enhance heart function and reduce and possibly reverse certain heart-related problems in Fabry disease [5]. Recent research indicates that ERT could bring about improvements in the size of the ventricle and cardiac function in individuals with Fabry disease and those with mild heart dysfunction. We are presenting a unique diagnostic finding for cardiomyopathy induced by Fabry’s disease to show the pattern of cardiac involvement in such cases.

## Case presentation

**Figure 1:**
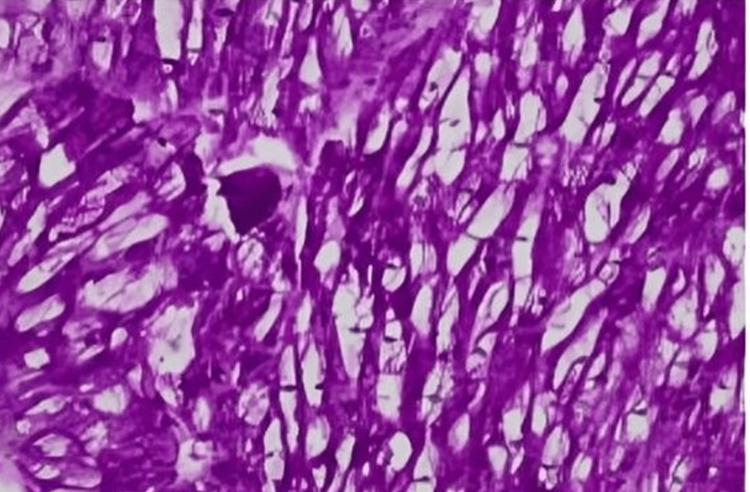
Section showing very unusual findings, which are diffuse, uniform, and severe myocytolysis (vacuolization of the cytoplasm of myocytes). HE X 200 (hematoxylin and eosin stain)

**Figure 2:**
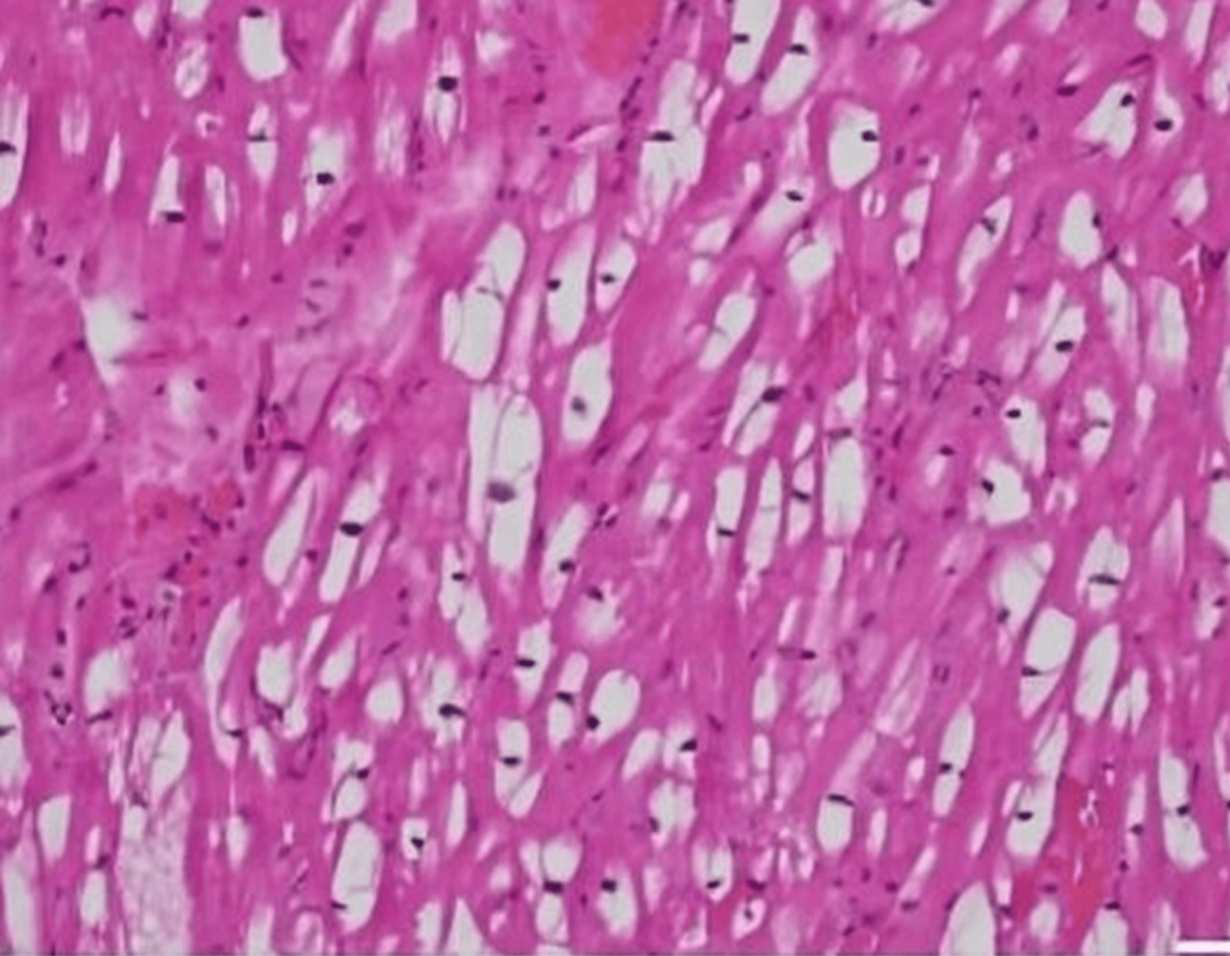
Special stain of PAS X 200 (periodic acid-Schiff) is positive

**Figure 3:**
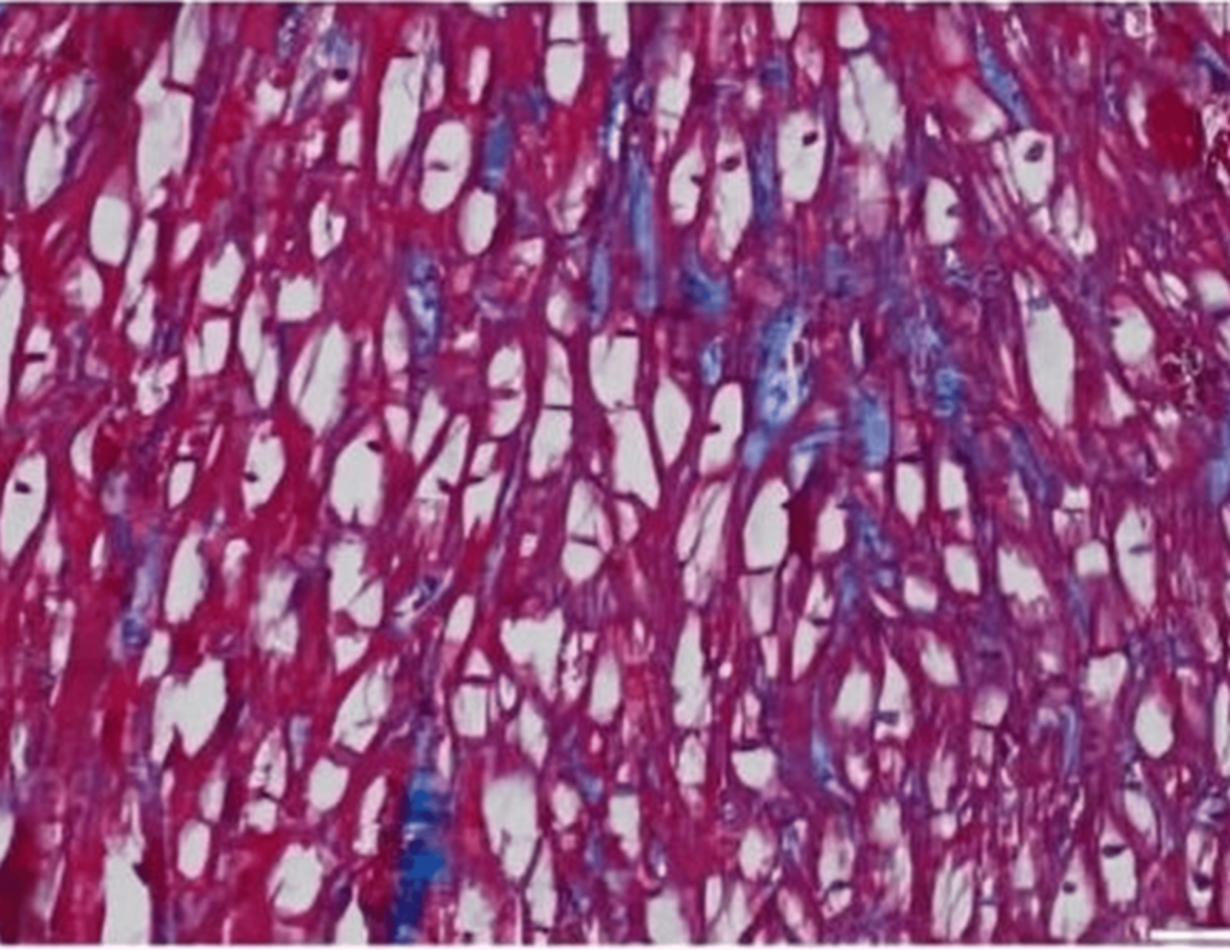
PAS (periodic acid-Schiff) with diastase X 200 is positive (diastase resistant)

**Figure 4:**
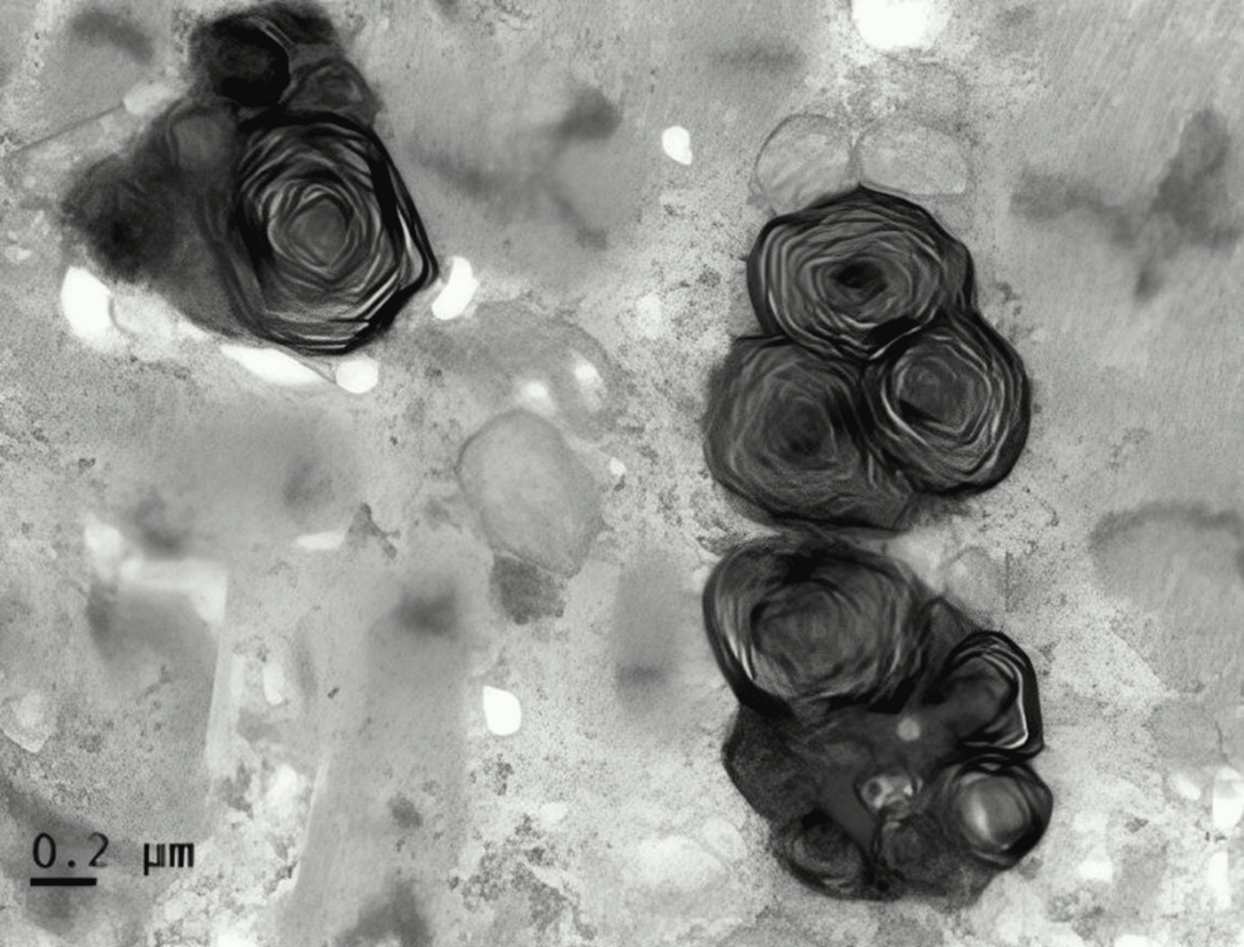
Electron microscope shows inclusion bodies described as myeloid and curvilinear bodies within cells, interstitial cells, and cardiomyocytes

**Figure 5:**
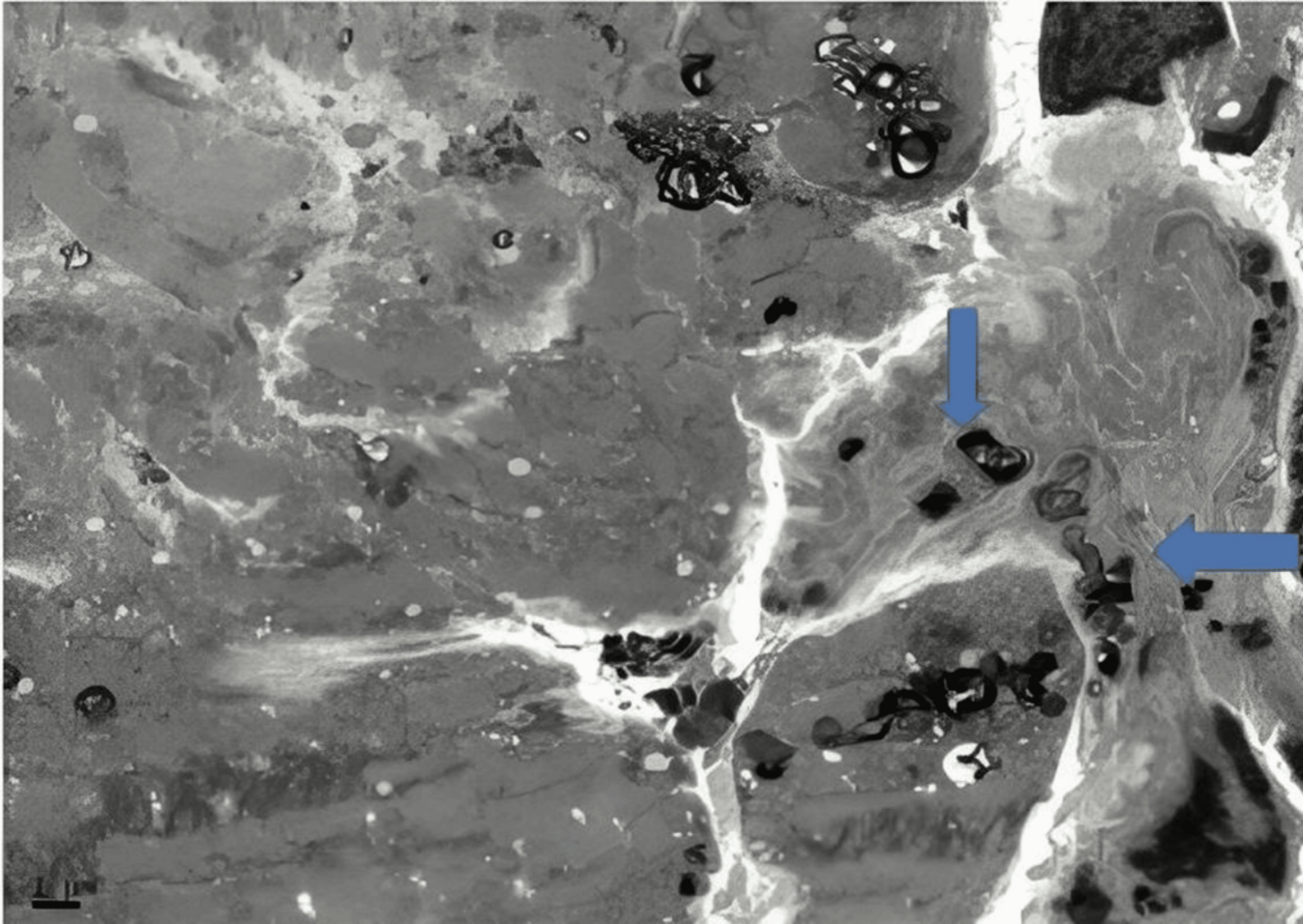
Electron microscope shows the myelin figures are also seen in interstitial spaces and vascular wall

A 57-year-old female diagnosed with end-stage renal disease, presumably due to hypertension, and currently on hemodialysis. She has a history of atrial fibrillation on amiodarone, along with a history of transient ischemic attack without sequelae. Her blood pressure is well controlled on carvedilol. Both parents and maternal grandmother had a history of end-stage renal disease (ESRD). During evaluation and workup for a possible kidney transplant, an echocardiogram showed an ejection fraction of 65-70% and severe LVH with an interventricular septum (IVS) of 1.7 cm. As a result of significantly low levels of alpha-galactosidase, globotriaosylceramide, and globotriaosylsphingosine enzymes which had been ordered by the clinical team, a concern for Fabry disease was raised, a genetic test was requested, and right heart catheterization with endomyocardial biopsy (EMB) was performed. On light microscopy, the H&E sections show very unusual findings, which are diffuse, uniform, and severe myocytolysis (vacuolization of the cytoplasm of myocytes) (Figure 1). Special stains of periodic acid Schiff stain (PAS) (Figure 2), and PAS with diastase are both positive (diastase resistant) (Figure 3), which rules out glycogen storage disease. Electron microscopy demonstrated that cardiomyocytes exhibit unusual ultrastructural features, including numerous myelin figures (laminated bodies) that replace the myocytes. These features correlate with the vacuolization of the cytoplasm observed under a light microscope. Additionally, there are intriguing inclusion bodies, described as myeloid and curvilinear bodies, found within cells, interstitial cells, and cardiomyocytes (Figure 4). The myelin figures are also seen in interstitial spaces and vascular walls (Figure 5). The unaffected myocytes have central nuclei and the interstitium contains capillaries and increased amounts of collagen fibrils with associated amorphous material. Our strategy, for the patient involves starting enzyme replacement treatment to halt organ system harm caused by Fabry’s disease. We aim to connect her with our genetics team at the Fabry’s Clinic. After optimizing the ERT, we will conduct updated tests for transplant evaluation, such, as chest X-ray (CXR), electrocardiogram (EKG), and echocardiogram. In addition to following up with the patient's relatives for genetic counseling.

## Discussion

Fabry’s disease, an uncommon genetic condition with diverse symptoms, was discovered by dermatologists Johannes Fabry and William Anderson in 1898 and is a progressive disorder that affects various organs in the body. It results from a lack of the enzyme α galactosidase A in lysosomes, leading to the buildup of lipids like globotriaosylceramide in cell types [6]. This condition mainly impacts males due to its X-linked inheritance pattern, with prevalence rates ranging from 1,40,000 to 1,117,000 in nations. However, females can also experience symptoms due to lyonization [1,3,5,7]. The patient in our case is, interestingly, female.

Individuals with Fabry’s disease display an array of symptoms affecting bodily systems such as the cardiovascular, renal, and nervous systems. Diagnosing this disorder can be complex, as its manifestations may mimic ailments like diabetes or hypertension [1,3,5], and other medical conditions. Our patient has a history of transient ischemic attack and end-stage renal disease, which may be part of the manifestation of Fabry's disease and definitely a cause of her heart abnormalities. The cardiovascular system plays a role in the morbidity and mortality associated with Fabry disease [1]. The heart problems associated with Fabry disease typically start before birth and result in LVH, which is observed among those patients without any other apparent cause. It has been seen in approximately 0.35% of those with hypertrophic cardiomyopathy [3]. Additionally, other heart-related complications include heart failure with preserved ejection fraction, which is a common symptom among individuals with Fabry disease [7], arrhythmia, and chest pain without evidence of coronary artery disease. Detecting the primary cause early is crucial because ERT can help lessen or even reverse some of the heart-related symptoms of Fabry disease [7]. Our patient has a preserved ejection fraction and severe LVH. Considering the impact on the heart, healthcare providers need to be vigilant for signs of Fabry disease in patients showing these symptoms, especially when traditional risk factors for cardiovascular problems are not present.

The progressive nature of Fabry disease and its impact on systems can make diagnosis challenging (summarized in Table 1), resulting in delays before a clear diagnosis is reached. However, advancements in enzyme testing, genetic analysis, and specialized clinical services for illnesses have led to an increase in the recognition of Fabry disease [7]. This has also allowed for more identification of asymptomatic individuals, emphasizing the importance of early detection of organ involvement and continuous monitoring to effectively manage Fabry's disease. Fortunately, in our case, the cardiac biopsy was very helpful in confirming the diagnosis. Ensuring a precise diagnosis, including biopsy, is crucial for initiating effective treatments for Anderson-Fabry disease (AFD). Biopsy plays a role in identifying the forms of this uncommon genetic condition. The results of the biopsy not only confirmed the diagnosis. It also offered important insights into the scope and seriousness of heart-related issues, which played a key role in determining the patient’s treatment [7,8].

**Table 1:** Fabry-related manifestations and common misdiagnosis Reference: [9,10] ESR: erythrocyte sedimentation rate

Fabry-related manifestations	Common misdiagnosis
Cardiovascular	Hypertrophic cardiomyopathy
Renal	End-stage kidney disease [9]
Pain, fever, and increased ESR	Rheumatic fever
Neurologic manifestations	Multiple sclerosis [10]
Systemic symptoms	Systemic lupus erythematous

Patients have been primarily receiving ERT as a part of their treatment approach since 2001 [8], and there are also emerging treatments being explored, such as chaperone therapy, plant-based enzymes, and gene therapy. ERT has shown results in reducing LVH and improving cardiac structure and function in adult patients [3]. Ongoing research is focusing on finding treatment options to manage aspects of this complex disorder. Detecting Fabry's disease promptly and correctly is essential not only for the patient but also for the family members [7]. When a person with Fabry’s disease is diagnosed, it can help identify family members affected by the condition, enabling intervention and potentially averting serious complications [2]. Since Fabry’s disease runs in families, diagnosing one individual can greatly impact the family, underscoring the need for a diagnostic strategy.

## Conclusions

Fabry disease, caused by mutations in the α GalA gene, leads to an accumulation of sphingolipids that can result in symptoms affecting the heart, kidneys, and nerves. In this case study, we present the history of a 57-year-old woman with advanced kidney disease who was diagnosed with cardiomyopathy linked to LVH. The findings from an EMB showed vacuolization, myeloid, and curvilinear bodies, as well as inclusions in endothelial cells, interstitial cells, and cardiomyocytes. Diagnosing Fabry disease can be challenging due to its similarity to other conditions. This case highlights the importance of utilizing biopsies for the diagnosis and treatment of Fabry's disease. Treatment options such as ERT are effective in managing the condition, and genetic testing should also be considered for family members.

## References

[1] O'Mahony C, Elliott P (2010). Anderson-Fabry disease and the heart. Prog Cardiovasc Dis.

[2] Teragaki M, Tanaka A, Akioka K, Lan HT, Nishi Y, Yamano T, Yoshikawa J (2004). Fabry disease female proband with clinical manifestations similar to hypertrophic cardiomyopathy. Jpn Heart J.

[3] Wong Wong, CY CY (2020). A rare and potentially treatable cause of left ventricular hypertrophy. Eur Heart J Cardiovasc Imaging.

[4] Weidemann F, Niemann M, Warnock DG, Ertl G, Wanner C (2011). The Fabry cardiomyopathy: models for the cardiologist. Annu Rev Med.

[5] Beck M, Ricci R, Widmer U (2004). Fabry disease: overall effects of agalsidase alfa treatment. Eur J Clin Invest.

[6] Kolter T, Sandhoff K (2006). Sphingolipid metabolism diseases. Biochim Biophys Acta.

[7] Baig S, Vijapurapu R, Alharbi F (2019). Diagnosis and treatment of the cardiovascular consequences of Fabry disease. QJM.

[8] Shah JS, Elliott PM (2005). Fabry disease and the heart: an overview of the natural history and the effect of enzyme replacement therapy. Acta Paediatr Suppl.

[9] Trachoo O, Jittorntam P, Pibalyart S (2016). Screening of Fabry disease in patients with end-stage renal disease of unknown etiology: the first Thailand study. J Biomed Res.

[10] Böttcher T, Rolfs A, Tanislav C (2013). Fabry disease - underestimated in the differential diagnosis of multiple sclerosis?. PLoS One.

